# The Effect of Platelet-Rich Fibrin in Preventing Alveolar Osteitis Following Mandibular Third Molar Surgery: A Systematic Review and Meta-Analysis

**DOI:** 10.7759/cureus.103522

**Published:** 2026-02-13

**Authors:** Gursimrat Brar, Asmita Sodhi, Gurmeet Singh Brar, Amandeep Kaur, Nitin Verma, Ramninder Bawa, Seema Gupta

**Affiliations:** 1 Department of Oral and Maxillofacial Surgery, Dasmesh Institute of Research and Dental Sciences, Faridkot, IND; 2 Department of Prosthodontics, Sodhi Dental Clinic and Implant Center, Faridkot, IND; 3 Department of Dentistry, Rubal’s Dental Clinic, Faridkot, IND; 4 Department of Oral and Maxillofacial Surgery, Government Dental College and Hospital, Amritsar, IND; 5 Department of Prosthodontics, Sri Guru Ram Das Institute of Dental Sciences and Research, Amritsar, IND; 6 Department of Orthodontics, Kothiwal Dental College and Research Center, Moradabad, IND

**Keywords:** alveolar osteitis, extraction, platelet-rich fibrin, prevention, third molar

## Abstract

Alveolar osteitis (AO) is a common and painful complication following mandibular third molar extraction, often resulting in delayed healing and increased postoperative morbidity. Platelet-rich fibrin (PRF) has been proposed as a biologically active adjunct that can enhance socket healing and stabilize postextraction blood clots. This systematic review and meta-analysis aimed to evaluate the effectiveness of PRF in preventing AO after mandibular third molar extraction. A comprehensive literature search was conducted in PubMed/MEDLINE, Scopus, Cochrane Central Register of Controlled Trials, and Google Scholar from database inception to the most recent update. Randomized controlled trials comparing PRF application with no PRF or conventional extraction care and reporting AO incidence were included. Data extraction and risk-of-bias assessment were performed independently using the Cochrane Risk-of-Bias 2 tool. A meta-analysis was conducted using a random-effects model, and effect estimates were expressed as risk ratios (RRs) with 95% confidence intervals (CIs). Sensitivity analysis for unmeasured confounding was performed using the E-value approach. Seven randomized controlled trials involving 776 extraction sites (388 PRF and 388 control) were included. The meta-analysis demonstrated a significant reduction in AO incidence with PRF application (RR = 0.33; 95% CI = 0.20-0.55; p < 0.05), corresponding to an approximately 67% reduction in relative risk. Heterogeneity among studies was negligible, and funnel plot inspection, along with Egger’s test, suggested a low likelihood of publication bias. The certainty of evidence for the primary outcome was rated as moderate. Sensitivity analysis yielded an E-value of 5.51, indicating the robustness of the observed effect to potential unmeasured confounding. Within the limitations of the available evidence, PRF appears to be a safe and effective adjunct for reducing the incidence of AO following mandibular third molar extraction. Further standardized, high-quality trials are warranted to optimize the clinical protocols and confirm the long-term benefits.

## Introduction and background

Alveolar osteitis (AO), commonly referred to as dry socket, is one of the most frequent postoperative complications following dental extractions, particularly after mandibular third molar extraction [1]. It is characterized by severe radiating pain, partial or complete loss of the intra-alveolar blood clot, exposed bone, and delayed wound healing, typically manifesting one to three days after extraction [2]. The reported incidence of AO ranges from 1% to 5% in routine extractions and may increase to 20% after surgical removal of impacted mandibular third molars [3,4]. Given its impact on patient discomfort, the need for repeated postoperative visits, and the increased healthcare burden, effective preventive strategies remain a clinical priority.

Conventional methods proposed for AO prevention include atraumatic surgical techniques, socket irrigation, topical antiseptics, antibiotics, medicated dressings, and antifibrinolytic agents [5]. However, none have achieved universal acceptance, largely due to inconsistent efficacy and potential adverse effects. In recent years, platelet-rich fibrin (PRF), a second-generation autologous platelet concentrate, has gained attention as a biologically driven preventive approach to manage peri-implantitis [6]. PRF provides a fibrin scaffold enriched with platelets, leukocytes, and growth factors that promote angiogenesis, epithelialization, and stabilization of the blood clot, mechanisms that are directly relevant to the pathophysiology of AO [6,7].

Several clinical trials have evaluated the use of PRF in third molar surgery, reporting favorable outcomes in terms of reduced postoperative morbidity and lower AO incidence [6,8,9]. Consequently, multiple systematic reviews have been published on this topic to date. However, a critical appraisal of the existing evidence reveals several important limitations. A prior systematic review combined studies evaluating the treatment of established AO with those assessing prevention [10], and another review focused primarily on secondary outcomes, such as pain, swelling, and healing [11]. Additionally, these reviews included heterogeneous platelet concentrates or lacked a quantitative synthesis of AO incidence, limiting their clinical interpretability. Importantly, no previous meta-analysis has exclusively and rigorously quantified the preventive effect of PRF on the incidence of AO following mandibular third molar surgery using a strictly defined comparative study design. Therefore, this systematic review and meta-analysis was designed to address this critical evidence gap. By including only controlled clinical trials that compared PRF with no PRF or standard extraction care and reported AO incidence as a primary outcome, this study provides a focused and methodologically robust synthesis of the preventive efficacy of PRF.

Aim and research question

This systematic review and meta-analysis aimed to evaluate the effect of PRF on the incidence of AO following mandibular third molar surgery. The research question was: Does the application of PRF at the time of mandibular third molar extraction reduce the incidence of AO compared with no PRF or conventional postoperative care?

## Review

Study design and reporting framework

This systematic review and meta-analysis was conducted in accordance with the Preferred Reporting Items for Systematic Reviews and Meta-Analyses 2020 guidelines [12]. The methodology was developed a priori to ensure transparency, reproducibility, and methodological rigor. This review specifically focused on evaluating the preventive effect of PRF on the incidence of AO following mandibular third molar surgery.

Population, intervention, comparison, outcomes, and study framework

The eligibility criteria were defined using the Population, Intervention, Comparison, Outcomes and Study framework as follows: Population (P) as patients undergoing surgical extraction of mandibular third molars with no clinical diagnosis of AO at baseline, Intervention (I) as placement of PRF in the extraction socket immediately after tooth removal, Comparison (C) as no PRF application, empty socket, or conventional postoperative care without a platelet concentrate, Outcomes (O) as the primary outcome was the incidence of AO, defined clinically by postoperative pain with partial or complete loss of the blood clot and exposed alveolar bone within one to seven days after extraction, Study design (S) as randomized controlled trials (RCTs) and prospective controlled clinical studies with a comparative design.

Eligibility criteria

Studies were included if they met the following criteria: 1) involved human participants undergoing mandibular third molar extraction, 2) evaluated PRF as a preventive adjunct applied at the time of surgery, 3) included a comparison group without PRF, 4) reported the incidence of AO with extractable numerical data, and 5) used a randomized or controlled prospective study design. Studies were excluded if they 1) evaluated PRF for the treatment of established AO, 2) lacked a comparator group, 3) used platelet concentrates other than PRF, 4) were case reports, case series, reviews, animal studies, or in vitro studies, or 5) did not provide sufficient data to calculate effect estimates.

Search strategy

A comprehensive electronic literature search was conducted across PubMed/MEDLINE, Scopus, Cochrane Central Register of Controlled Trials (CENTRAL), and Google Scholar from database inception to the most recent update (December 15, 2025). The search strategy combined Medical Subject Headings and free-text terms related to PRF, AO, dry socket, and third molar surgery. Boolean operators (“AND,” “OR”) were used to refine the search (Table 1). Additionally, the reference lists of the included studies and relevant reviews were manually screened to identify any potentially eligible articles not captured by the electronic search.

**Table 1:** Databases searched and electronic search strategy PRF: platelet-rich fibrin

Database	Search terms used
PubMed/MEDLINE	(“Platelet-Rich Fibrin” OR PRF) AND (“Alveolar Osteitis” OR “Dry Socket”) AND (“Mandibular Third Molar” OR “Third Molar Surgery” OR “Wisdom Tooth Extraction”)
Scopus	TITLE-ABS-KEY (“platelet rich fibrin” OR PRF) AND (“alveolar osteitis” OR “dry socket”) AND (“mandibular third molar” OR “third molar surgery”)
Cochrane CENTRAL	(“platelet rich fibrin”) AND (“alveolar osteitis” OR “dry socket”) AND (“third molar”)
Google Scholar	“Platelet rich fibrin” AND “alveolar osteitis” AND “mandibular third molar”
Manual search	Reference lists of included studies and relevant systematic reviews

Study selection

All retrieved records were imported into reference management software, and duplicates were eliminated. Two reviewers independently screened the titles and abstracts to identify potentially eligible studies. The full-text articles of the shortlisted studies were then independently assessed against the inclusion and exclusion criteria. Disagreements were resolved through discussion and consensus.

Data extraction

Data extraction was performed independently by two reviewers using a standardized data extraction form. The following information was collected: author and year of publication, country, study design, sample size, participant demographics, surgical site, PRF preparation protocol, comparator details, follow-up duration, diagnostic criteria for AO, and number of AO events in each group. When required, the corresponding authors were contacted for clarification of missing or unclear data.

Risk-of-bias assessment

The risk of bias of the included RCTs was assessed using the Cochrane Risk-of-Bias 2 (RoB 2) tool [13], which evaluates bias arising from randomization, deviations from intended interventions, missing outcome data, outcome measurement, and selective reporting. Each study was classified as having a low risk, some concerns, or a high risk of bias.

Statistical analysis

Meta-analysis was performed using Review Manager (RevMan, version 5.4, The Cochrane Collaboration, London, United Kingdom). The primary outcome, incidence of AO, was analyzed as a dichotomous variable, and pooled effect estimates were expressed as risk ratios (RRs) with corresponding 95% confidence intervals (CIs). A random-effects model was applied to account for potential clinical and methodological heterogeneity among the included studies. Statistical heterogeneity was assessed using the chi-square (χ²) test and quantified with the I² statistic, with values greater than 50% considered indicative of substantial heterogeneity. The between-study variance (τ²) was also estimated, and a prediction interval was calculated to reflect the expected range of effects in future comparable studies. Sensitivity analyses were conducted to evaluate the robustness of the pooled estimates, including leave-one-out analysis and E-value-based sensitivity analysis to assess the potential impact of unmeasured confounding. Publication bias was evaluated through visual inspection of funnel plot asymmetry and Egger’s regression test, while acknowledging the limitations associated with the small number of included studies. A two-sided p value of <0.05 was considered statistically significant.

Results

Study Selection

**Figure 1:**
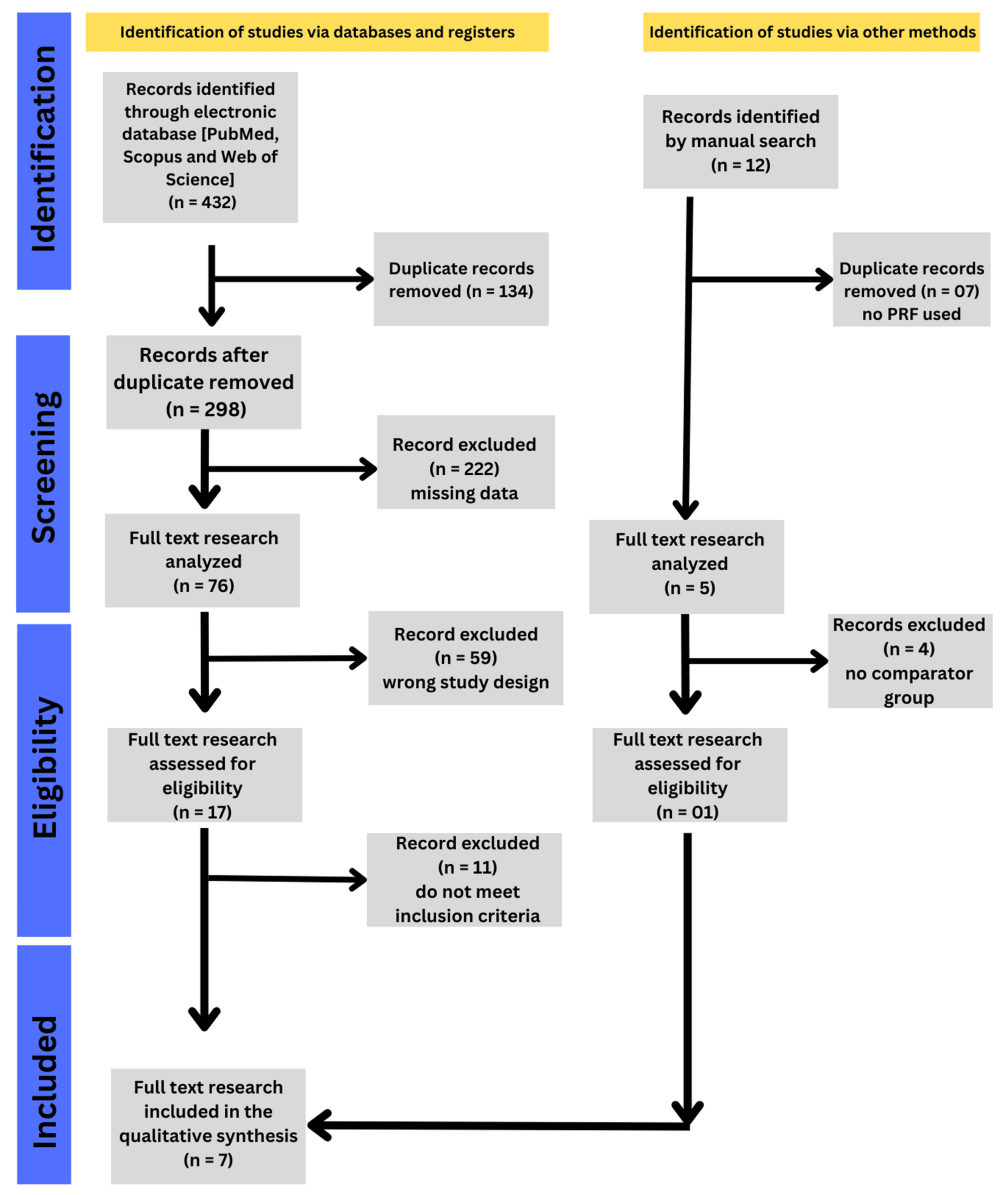
PRISMA flow diagram of study selection PRF: platelet-rich fibrin Image credit: This is an original image created by the author Gursimrat Brar

The electronic database search yielded a total of records across PubMed/MEDLINE, Scopus, Cochrane CENTRAL, and Google Scholar databases. After removing duplicates, the titles and abstracts were screened for relevance. Full-text evaluation was performed for potentially eligible studies. Following the application of the predefined inclusion and exclusion criteria, seven studies were found to be eligible [14-20] and were included in the qualitative and quantitative syntheses (Figure 1). Studies evaluating PRF for the treatment of established AO, those lacking a comparator group, and those using platelet concentrates other than PRF were excluded.

Characteristics of Included Studies

The seven included RCTs were published between 2014 and 2023 and were conducted in Iran, Turkey, Pakistan, Egypt, and Lithuania. Three studies employed a split-mouth design, and four employed a parallel-group design. A total of 776 mandibular third molar extraction sites were evaluated, with 388 sites allocated to the PRF group and 388 sites to the control group. Across all studies, PRF was prepared using standardized centrifugation protocols (2,700-3,000 rpm for 10-12 minutes) and was placed immediately into the extraction socket following tooth removal. The primary outcome assessed in all studies was the incidence of AO, evaluated clinically between the third and seventh postoperative days. The detailed study characteristics are summarized in Table 2.

**Table 2:** Characteristics of studies included in the meta-analysis PD: parallel study-design; SM: split-mouth; RCT: randomized controlled trial; AO: alveolar osteitis; PRF: platelet-rich fibrin; rpm: revolutions per minute

Study	Country	Year	Study design	Study group	Control	Age (years)	Male/female	PRF preparation	Outcome	Follow-up
Eshghpour et al. [14]	Iran	2014	RCT SM	78	78	25.09 ± 4.25	33/45	2,800 rpm, 12 minutes	AO incidence	Seventh postoperative day
Unsal and Erbasar [15]	Turkey	2018	RCT SM	50	50	23.96	17/33	3,000 rpm, 10 minutes	AO incidence	Seventh postoperative day
Asif et al. [16]	Pakistan	2023	RCT PD	90	90	41.35 ± 9.87	90/90	3,000 rpm, 10 minutes	AO incidence	Third postoperative day
Iqbal et al. [17]	Pakistan	2023	RCT PD	85	85	24.28 ± 3.7	87/83	3,000 rpm, 10 minutes	AO incidence	Third postoperative day
Al-Hamed et al. [18]	Egypt	2017	RCT PD	25	25	25.24 ± 7.04	13/34	3,000 rpm, 10 minutes	AO incidence	Seventh postoperative day
Asutay et al. [19]	Turkey	2017	RCT SM	30	30	20.32	6/24	2,700 rpm, 12 minutes	AO incidence	Seventh postoperative day
Daugela et al. [20]	Lithuania	2018	RCT SM	30	30	22.76	12/18	2,800 rpm, 12 minutes	AO incidence	Seventh postoperative day

Risk-of-Bias Assessment

**Figure 2:**
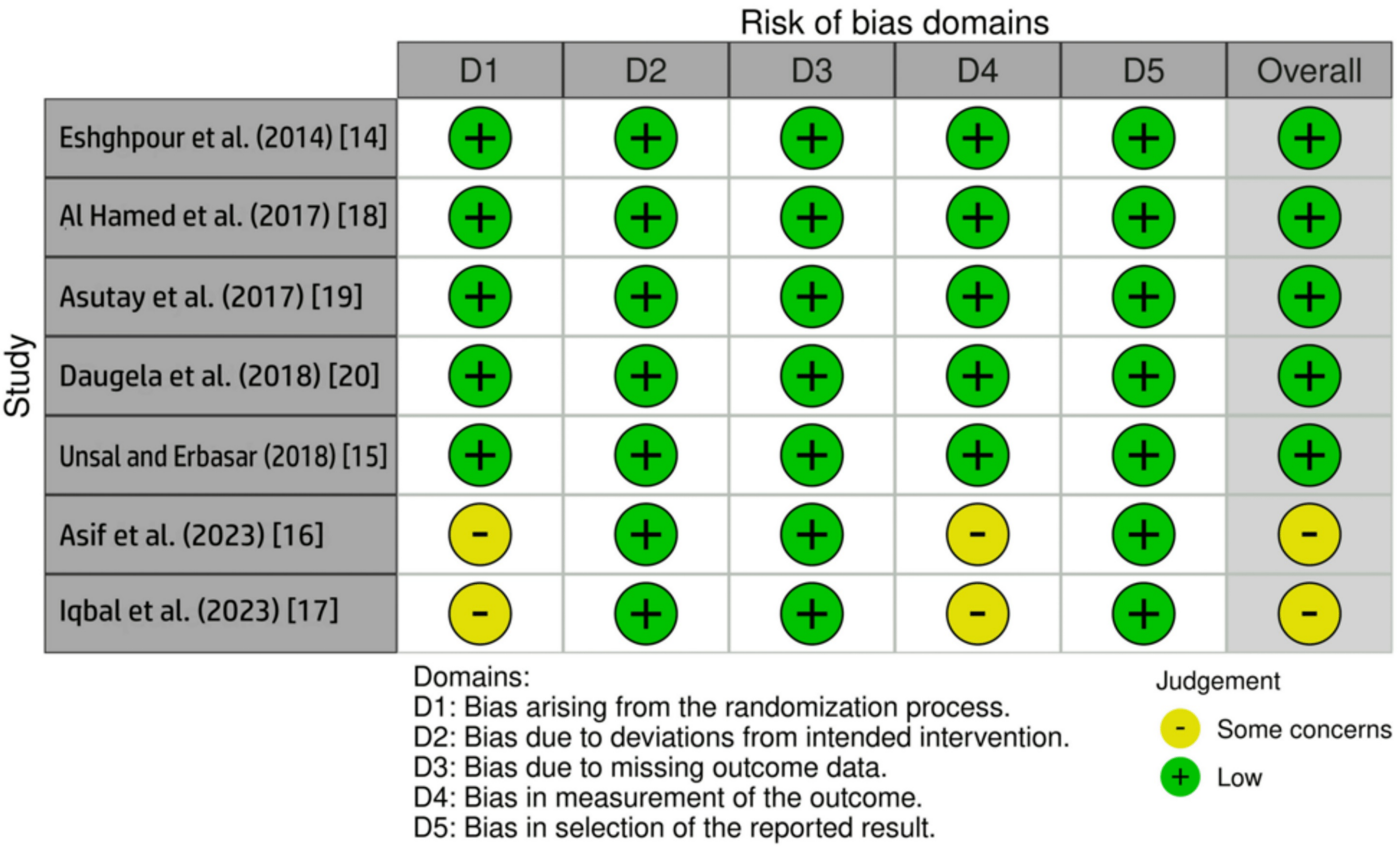
Risk-of-bias assessment of included studies using the Cochrane risk-of-bias 2 tool Green symbols indicate low risk of bias, while yellow symbols indicate some concerns. Overall risk-of-bias judgments are shown in the final column Image credit: This is an original image created by the author Gursimrat Brar

The risk of bias was assessed using the Cochrane RoB 2 tool across five domains. Five studies [14,15,18-20] were judged to be at low risk of bias across all domains, reflecting appropriate randomization, standardized surgical procedures, complete outcome data, and a clear outcome assessment. Two studies [16,17] were judged to have some concerns, primarily related to insufficient reporting of allocation concealment and outcome assessor blinding. No study was judged to have a high risk of bias. An overall summary of the risk-of-bias assessment is presented in Figure 2.

Meta-Analysis of AO Incidence

**Figure 3:**
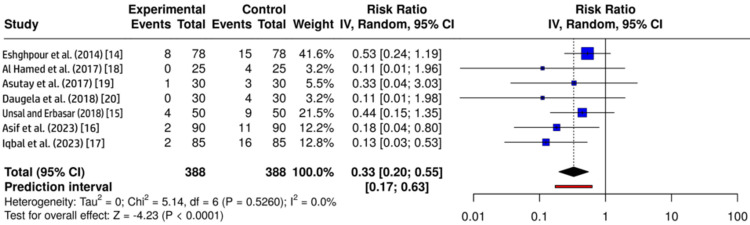
Forest plot showing the effect of platelet-rich fibrin on the incidence of alveolar osteitis following mandibular third molar extraction The size of the square represents the weight of each study in the meta-analysis, and horizontal lines indicate 95% CIs. The diamond represents the overall pooled effect estimate CI: confidence interval; df: degrees of freedom Image credit: This is an original image created by the author Gursimrat Brar

All seven studies provided dichotomous data for AO and were included in the meta-analysis. A random-effects model was applied using the inverse variance method. The pooled analysis demonstrated a statistically significant reduction in the incidence of AO in the PRF group compared to that in the control group (RR = 0.33; 95% CI = 0.20-0.55; p < 0.05). This indicates that PRF use reduced the AO risk by approximately 67% after mandibular third molar extraction. The assessment of heterogeneity showed no notable between-study variability, with a consistent direction and magnitude of the effect across all included trials (Figure 3).

Publication Bias

**Figure 4:**
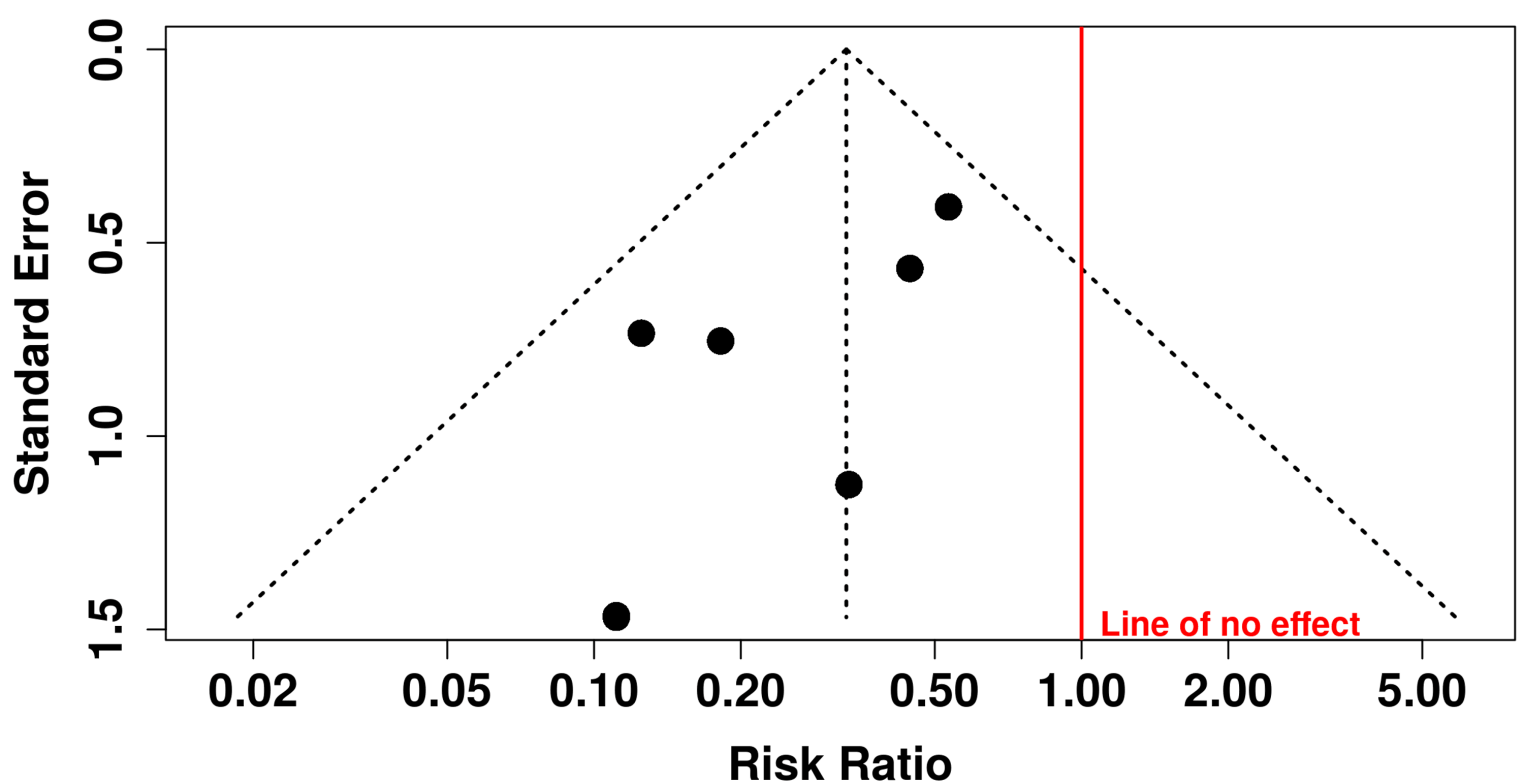
Funnel plot for assessment of publication bias Funnel plot showing the distribution of individual study effect estimates (risk ratios) against their standard errors for the included randomized controlled trials evaluating platelet-rich fibrin in the prevention of alveolar osteitis. The vertical red line represents the line of no effect (risk ratio = 1.0). The dashed lines indicate the pseudo 95% confidence limits Image credit: This is an original image created by the author Gursimrat Brar

**Figure 5:**
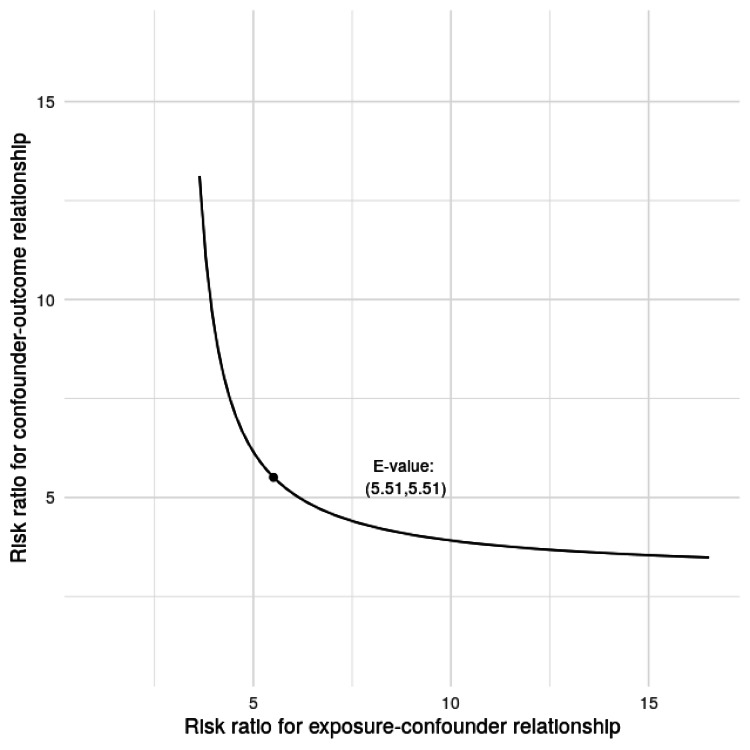
E-value-based sensitivity analysis for unmeasured confounding The marked point corresponds to the calculated E-value of 5.51 for both the point estimate and the lower bound of the confidence interval Image credit: This is an original image created by the author Gursimrat Brar

Publication bias was assessed visually using a funnel plot and statistically using Egger’s regression test. The funnel plot showed no marked asymmetry. Egger’s test did not indicate significant small-study effects (intercept = -1.55; 95% CI = -2.75 to -0.35; p = 0.053), suggesting a low likelihood of publication bias (Figure 4). A sensitivity analysis was conducted using the E-value approach to assess the robustness of the association between PRF application and the incidence of AO to potential unmeasured confounding. The pooled effect estimate demonstrated an E-value of 5.51 for both the point estimate and the lower bound of the CI. The E-value plot illustrates the trade-off between the strength of the exposure-confounder and confounder-outcome associations required to nullify the observed effect. Overall, this analysis supports the robustness of the meta-analytic findings and suggests that the observed reduction in AO with PRF use is unlikely to be explained solely by confounding factors (Figure 5).

Certainty of Evidence (Grading of Recommendations Assessment, Development, and Evaluation)

According to the Grading of Recommendations Assessment, Development, and Evaluation approach [21], the certainty of evidence for the primary outcome (incidence of AO) was rated as moderate. The quality of evidence was evaluated across five domains: risk of bias, inconsistency, indirectness, imprecision, and publication bias assessment. Although all included studies were RCTs, the certainty was downgraded due to concerns regarding risk of bias and imprecision related to variability in follow-up timing and PRF preparation protocols. Overall, the findings indicate that PRF probably reduces the incidence of AO following mandibular third molar extraction (Table 3).

**Table 3:** GRADE analysis ¹Downgraded due to some concerns regarding risk of bias and clinical heterogeneity in PRF preparation protocols CI: confidence interval; GRADE: Grading of Recommendations Assessment, Development, and Evaluation; RCT: randomized controlled trial; PRF: platelet-rich fibrin

Outcome	Number of studies (extraction sites)	Risk ratio (95% CI)	Anticipated absolute effects	Certainty of the evidence (GRADE)	Plain language summary
Incidence of alveolar osteitis (critical)	7 RCTs (776)	0.33 (0.20-0.55)	Control: 100 per 1,000 PRF: 33 per 1,000 (20-55)	⊕⊕⊕◯ Moderate¹	PRF probably reduces alveolar osteitis by about two-thirds after mandibular third molar surgery

Secondary Outcomes

The secondary outcomes reported across the included studies primarily included postoperative pain, soft tissue healing, swelling, trismus, and periodontal probing depth adjacent to the second molar. Owing to substantial heterogeneity in outcome definitions, assessment tools (visual analog scales, verbal rating scales, and clinical scoring systems), follow-up intervals, and reporting formats, a quantitative synthesis of secondary outcomes was not feasible. Qualitative assessment indicated that most studies reported lower postoperative pain scores in the PRF group, particularly during the early postoperative period (third to seventh postoperative days). Improvements in soft-tissue healing and socket conditions have also been noted in several studies. The findings related to swelling and trismus were inconsistent, with some trials reporting no significant intergroup differences. Periodontal probing depth measurements, when reported, were comparable between the PRF and control groups at follow-up. Overall, while the secondary outcomes suggest potential benefits of PRF in early postoperative recovery, the evidence remains inconclusive owing to methodological heterogeneity.

Discussion

AO remains one of the most common and distressing complications following mandibular third molar surgery, with a multifactorial etiology involving local trauma, fibrinolysis, microbial activity, and impaired clot stability. The present systematic review and meta-analysis evaluated the preventive effect of PRF on the incidence of AO after mandibular third molar extraction. A pooled analysis of seven RCTs demonstrated that PRF application significantly reduced the incidence of AO, with an overall risk reduction of approximately two-thirds. These findings provide moderate-certainty evidence supporting the adjunctive use of PRF in third molar surgery.

PRF helps prevent AO primarily by stabilizing postextraction blood clots and enhancing early wound healing within the socket. The fibrin matrix of PRF acts as a biological scaffold, mechanically protecting the clot from dislodgement and reducing exposure of the alveolar bone, which is a key event in AO pathogenesis. In addition, PRF releases a sustained concentration of growth factors, such as platelet-derived growth factor, transforming growth factor-β, and vascular endothelial growth factor, which promote angiogenesis, fibroblast migration, and epithelialization of the extraction socket. The presence of leukocytes and cytokines in PRF also contributes to local immunomodulation, limiting bacterial colonization and excessive fibrinolytic activity. Together, these effects counteract clot breakdown, reduce inflammation, and accelerate soft-tissue closure, thereby lowering the risk of AO after tooth extraction [7,10,11,22].

The consistency of the effect across studies, despite variations in study design (split-mouth vs. parallel-group), follow-up timing, and PRF preparation protocols, strengthens the validity of the findings. Notably, heterogeneity was negligible, and the direction of the effect was uniform across all the included trials. This suggests that the beneficial effect of PRF on AO prevention is robust and reproducible in different clinical settings and populations. In most studies, PRF has been used for the treatment of AO after third molar extraction, and beneficial effects have been observed [10,11,23]. Furthermore, sensitivity analysis using the E-value approach indicated that a very strong unmeasured confounder would be required to fully explain the observed association, further supporting the credibility of the results.

The risk-of-bias assessment showed that most of the included trials were at low risk of bias across all domains. Two studies were judged to have some concerns, primarily related to insufficient reporting of allocation concealment and outcome assessor blinding. However, these concerns are unlikely to substantially influence the primary outcome, as AO diagnosis is largely based on clinical criteria that are relatively objective and standardized across studies. Importantly, no study was judged to have a high risk of bias, which enhances confidence in the pooled estimates.

Secondary outcomes, including postoperative pain, swelling, trismus, soft tissue healing, and periodontal probing depth, were inconsistently reported across studies. Qualitative synthesis suggested that PRF may reduce early postoperative pain, particularly during the first postoperative week. Zwittnig et al. [24] reported a significant reduction in swelling with PRF placement after third molar extractions; however, no significant changes were noticed in clinical attachment loss, pain, or trismus. In contrast, another study reported a significant improvement in pain with PRF [25]. Another systematic review reported that both PRF and platelet-rich plasma positively influenced healing after third molar extraction [26].

However, our findings related to swelling and trismus were inconsistent, and the periodontal outcomes were largely comparable between the PRF and control groups. Due to heterogeneity in outcome measures and reporting formats, a quantitative synthesis of secondary outcomes was not feasible. These findings suggest that while PRF may confer additional postoperative benefits beyond AO prevention, the evidence is inconclusive. The findings of this review are consistent with those of previous clinical and experimental studies reporting a reduced incidence of AO following PRF application [14-20]. Earlier trials have highlighted the role of PRF in enhancing socket healing [26] and reducing postoperative complications, particularly in high-risk patients, such as smokers or those undergoing difficult extractions. The present meta-analysis strengthens this evidence base by providing a quantitative estimate of the effect derived exclusively from RCTs.

Clinical Implications

From a clinical perspective, the use of PRF is a simple, autologous, and cost-effective adjunctive measure for reducing the risk of AO following mandibular third molar surgery. PRF preparation is minimally invasive, does not require anticoagulants or exogenous additives, and can be readily integrated into the routine surgical workflow. Given the substantial reduction in AO incidence observed in this review, clinicians may consider PRF application, particularly in patients at higher risk of developing AO, such as those undergoing traumatic extractions or with known local risk factors. The potential reduction in postoperative morbidity may also translate to improved patient satisfaction and a reduced need for additional postoperative interventions.

Limitations

Several limitations should be considered when interpreting the findings of this study. First, variability in PRF preparation protocols, including centrifugation speed and duration, may influence the biological properties of PRF and contribute to this clinical heterogeneity. Second, the follow-up periods for AO assessment ranged from the third to the seventh postoperative day, which may have affected the reported incidence rates. Third, although all the included studies were RCTs, some had relatively small sample sizes, which may limit the statistical power for secondary outcomes. Finally, heterogeneity in the assessment and reporting of secondary outcomes precluded quantitative synthesis, limiting the conclusions regarding postoperative pain, swelling, and periodontal parameters.

Future Recommendations

Future research should focus on well-designed, adequately powered RCTs with standardized PRF preparation protocols and uniform diagnostic criteria for AO. Longer follow-up periods would help clarify the impact of PRF on periodontal healing and other late-postoperative outcomes. Additionally, subgroup analyses evaluating the effectiveness of PRF in high-risk populations, such as smokers and patients with complex impactions, would be clinically valuable. Comparative studies assessing PRF against other preventive modalities for AO may further inform evidence-based clinical decision-making.

## Conclusions

This systematic review and meta-analysis of seven RCTs provided moderate-certainty evidence that the application of PRF at the time of mandibular third molar extraction significantly reduces the incidence of AO. The pooled analysis demonstrated an approximately 67% relative reduction in risk of AO in sockets treated with PRF compared with conventional extraction care. The observed effect was consistent across studies, with minimal heterogeneity and no evidence of significant publication bias, and was robust to sensitivity analysis for unmeasured confounding. Given its autologous nature, biological plausibility, and ease of clinical application, PRF represents a safe and effective adjunctive preventive strategy for reducing AO following mandibular third molar surgery. However, variability in PRF preparation protocols and follow-up assessment underscores the need for standardized, well-designed randomized trials to further refine clinical guidelines and optimize its preventive use.
